# Evaluating Perspectives Toward Fertility and Motherhood Among Adolescent Females From Multi-country Backgrounds: A Descriptive, Cross-Sectional Study

**DOI:** 10.7759/cureus.82020

**Published:** 2025-04-10

**Authors:** Howieda Fouly, Ayat M Omar, Sadeq Al-Fayyadh, Nuran Aydin, Shatha Dababesh, Intisar Alsheikh, Mervat A Sayed, Zahra Abbas, Amira Elhoufy, Khaled Saad

**Affiliations:** 1 Health Sciences: Nursing, Higher Colleges of Technology, Ras Al Khaimah, ARE; 2 Maternity, Obstetrics, and Gynecology, Faculty of Nursing, Assiut University, Asyut, EGY; 3 Nursing, Faculty of Applied Medical Sciences, Jouf University, Qurayyt, SAU; 4 Maternity and Neonatal Health: Nursing, College of Nursing, Fayoum University, Fayoum, EGY; 5 Adult Nursing, College of Nursing, University of Baghdad, Baghdad, IRQ; 6 Critical Care Nursing, Istanbul Medipol University, Istanbul, TUR; 7 College of Nursing, Jouf University, Qurayyt, SAU; 8 Community Health Nursing: Nursing, University of Jeddah, Jeddah, SAU; 9 Community Health Nursing, Faculty of Nursing, Fayoum University, Fayoum, EGY; 10 College of Nursing, University of Basrah, Basrah, IRQ; 11 Community Health Nursing, College of Nursing, Faculty of Nursing, Al-Darb Jazan University, Jazan, SAU; 12 Community Health Nursing, Faculty of Nursing, Assiut University, Asyut, EGY; 13 Pediatrics, Assiut University, Asyut, EGY

**Keywords:** adolescents, cross-sectional study, female, fertility, human, motherhood

## Abstract

Background

Most students have essential perspectives on parenthood, which may vary based on education or cultural background. Negative perspectives reflect a serious gap in reproductive health education among future parents and may lead to delaying parenthood, increasing the risk of infertility or fetal loss.

Aim

This study aimed to assess students’ perspectives on fertility-related issues and motherhood across multi-country backgrounds.

Methods

A descriptive, cross-sectional design was conducted from Spring 2021 to Spring 2022 among voluntary participants focused on the fertility and motherhood perspectives of health sciences colleges in six participating countries (Egypt, Iraq, Jordan, Saudi Arabia, Sudan, and Turkey). The responses were collected conveniently by a self-administered questionnaire consisting of 31 items in 5 sections, adapted from a published Swedish study after testing for validity and reliability and analyzed using SPSS version 22 (IBM Corp., Armonk, NY, US) including 1,371 students from an online hosting site.

Results

Almost three-quarters of students have poor perspectives toward fertility concepts. There are significant relationships between students’ characteristics (independent variables) and their perspectives (dependent variable) toward motherhood. A high positive correlation between students’ decision to have children’s factors, their perspectives regarding cryopreservation, and motherhood with (*r* = 0.527, *p* <0.01), (*r* = 0.533, *p* <0.01), and (*r* =0.406, *p* <0.01). Students’ perspectives toward fertility reflect high frequency positive (*F* = 12.080, *p*< 000).

Conclusion

There is a positive correlation between participants’ decision to have children, cryopreservation perspectives, and motherhood with a high-frequency positive effect on students’ perspectives toward fertility issues. The study recommends that nurses provide necessary informed guidance and support to young females and males who are making fertility-related decisions.

## Introduction

Background

A majority of students held essential perspectives on parenthood, emphasizing the importance of a stable relationship, maturity, shared responsibilities with a partner, financial stability, reliable childcare, and a flexible and supportive career [1,2]. However, in students from Western universities, fertility perspectives for future motherhood revealed that 9 out of 10 participants desire to have children and regard parenthood as a highly significant aspect of their future lives [3]. In addition, avoidance of motherhood is common among individuals with irregular incomes and those involved with multiple partners. These factors contribute to delays in having the first child, ultimately resulting in unintended declines in fertility or even infertility [4,5]. In European countries, the majority of fertility awareness studies predominantly involving university students indicate that over half of the respondents plan to have children after the age of 35 [6,7]. Furthermore, a significant number of young adults underestimate the impact of a woman’s advanced age on fecundity [7]. Moreover, fertility preservation technology like cryopreservation was initially developed for medical reasons, although the accessibility of different reproductive techniques recently became an option for all fertile women and is called social oocyte freezing for non-medical reasons [8].

Education provides opportunities and amplifies the influence of marginalized groups, especially women in society [9]. However, in Middle Eastern and North African countries, the mechanism through which education impacts fertility remains unexplored. The reasons behind the correlation between education and reduced fertility are debated, with limited evidence available on the effects of education on fertility [10].

In Egypt, the "Survey of Young People in Egypt" in 2015 confirmed an increase in intention to have children among young unmarried people between 2009 and 2014 [11]. However, recent evidence shows a decline in fertility to 3.1 in 2018 [12], highlighting the importance of identifying factors that contribute to the increased fertility among the young Egyptian population [13]. In Saudi Arabia, the General Authority for Statistics (2016) reported a significant decline in the fertility rate. The average number of live births for Saudi women who have passed climacteric age (45-49) was 4.9, whereas the current average number of live births per Saudi woman decreased to 2.4 [14]. In a descriptive cross-sectional survey study conducted in Iraq targeting medical and non-medical Iraqi students, it was found that it is crucial to raise students’ awareness of fertility periods [2]. Additionally, Omran et al. assessed university students’ attitudes toward family planning programs and concluded that over one-quarter of 1900 students surveyed held negative attitudes toward these programs. This negative attitude highlights a significant gap in reproductive health education among future parents [15].

Recently, survey studies in Jordan indicate that fertility rates remained at around 3.8 children per woman from 1998 to 2012, after which they began to decline. This decline in fertility is most pronounced among women aged 25-29, likely due to postponing childbirth or forgoing motherhood altogether [16]. Conversely, in Sudan, no studies explore students’ perspectives on fertility and motherhood. Factors influencing fertility among Sudanese women are delayed marriage, increased migration rates, higher average age at the first marriage, women’s education attainment, and women's employment status [17].

A study in Turkey revealed that most university students possess considerable fertility knowledge and express a desire to have children. However, they tend to postpone childbearing to older ages. In cases of future infertility, the majority would prefer to utilize artificial reproductive technology for achieving parenthood [18].

Schmidt et al. reported that delaying parenthood increases the risk of infertility and fetal loss, including spontaneous abortions, ectopic pregnancies, and stillbirths. Numerous surveys have examined students’ intentions regarding family formation and their awareness of age-related impacts on fecundity [19]. Generally, over 90% of adults in their twenties who do not yet have children are expecting to become parents in the future [20]. A recent study in Denmark in 2016 highlighted the importance of addressing the potential association between fertility knowledge and parenthood planning [1].

Cryopreservation as a method of fertility preservation requires knowledge and understanding of associated evidence and the potential challenges of fertility conservation. Previous studies have indicated a lack of comprehensive understanding with regard to fertility [21,22].

Evidence from previous studies demonstrates that many healthcare providers do not adequately disclose the risks of treatment-related delayed fertility despite established guidelines on fertility counseling [23,24]. This results in a significant gap in fertility care among adolescents and young adults [25]. Consequently, delaying future fertility or motherhood is a prevalent concern among age groups. It is essential to investigate this concern further, especially determining the specific contributions of nurses to fertility counseling, which remain poorly identified. In addition, to enhance the understanding of family formation and fertility awareness, our study aims to assess students’ perspectives on fertility across multi-country backgrounds.

The research questions employed in this study are: What are the perspectives of participants toward motherhood across multi-country backgrounds?; What are the factors affecting participants’ decisions toward motherhood across multi-country backgrounds? What are the participants’ perspectives toward fertility issues across multi-country backgrounds?

## Materials and methods

Operational definition of fertility issues

In the current study, fertility issues refer to individuals' attitudes and concerns related to their perspectives on motherhood, their reproductive intentions, and their considerations regarding fertility preservation methods such as cryopreservation.

Study design

This study adopts a descriptive, cross-sectional design.

Study settings

This study was conducted in six countries: Egypt, Iraq, Jordan, Saudi Arabia, Sudan, and Turkey. Selection and participation were done through a voluntary approach focusing on the perspectives of fertility and motherhood among students in health sciences colleges, including Nursing, Medicine, Physiotherapy, and Medical Laboratories, of the participating countries, which consisted of first to sixth year and internship.

Study sample and sampling techniques

A convenience sample of 1,371 students was selected from various health sciences colleges across different countries. This sampling technique was based on inclusion criteria for the population from all the participating universities. To be eligible, participants had to (1) be enrolled in a bachelor program of the participating colleges, (2) be a full-time student, and (3) hold nationality in the country of residence. The exclusion criteria included residing in a country not included in the study. The sample was collected through online forms, with links distributed and shared by each author according to their respective countries.

The sample size of 1,371 was analyzed using G*Power version 3.1.9.2 software (Heinrich Heine University, Düsseldorf) for post hoc statistical power analysis for determining the achieved power. The analysis revealed that the statistical power for this study was 95%, with a medium effect size at the 5% level of significance, indicating adequate power. The study includes independent, dependent, and control variables.

Independent Variables

Multi-country backgrounds: This variable represents the different contexts related to fertility issues across multiple countries from which adolescent females are drawn.

Dependent Variables

Perspectives toward fertility: This variable includes the attitudes, beliefs, knowledge, and intentions of adolescent females regarding fertility. It encompasses their desires for parenthood, perceptions of the ideal timing for childbearing, attitudes toward family planning methods, and cultural influences on reproductive decisions.

Perspectives toward motherhood: This variable includes the attitudes, beliefs, and expectations of adolescent females regarding motherhood, including their views on motherhood and its associated challenges.

Control Variables

Age: This includes females aged from 18 to 30 years, as age may influence perspectives toward fertility and motherhood.

Education level: The level of education attained by adolescent females may impact their understanding of fertility and motherhood, as well as their ability to make informed decisions when it comes to reproductive health.

Socioeconomic status: Factors such as income, occupation, and access to healthcare services may influence adolescent females’ perspectives toward fertility and motherhood.

Ethical considerations

The study protocol was reviewed and approved by the Institutional Review Board of the Jouf University, College of Applied Medical Sciences in Saudi Arabia on November 24, 2020 (No. 3/42/27882). The participating universities reviewed and approved the protocol before its implementation with the students. This study is nonexperimental and was conducted with permission and approval from all the participating countries.

The online survey included a written informed consent form and was collected electronically from each respondent, who provided adequate information about the study. The study ensured the anonymity and confidentiality of all the participants by not disclosing their identities in the questionnaire. The consent form included a statement reassuring participants that the teacher-student relationship would not be affected by their participation or withdrawal from the study at any time. All the participants were informed of their right to terminate or withdraw from the study without any consequences.

After the study proposal was approved, regular communication channels, such as emails and the ResearchGate platform/website, were utilized for inviting coauthors from different countries. A self-administered questionnaire was used for data gathering between Spring 2021 and Spring 2022. A web-based online tool, hosted on a designated site, was forwarded as a link to the study participants for easy access.

A self-administered questionnaire, originally adapted from a Swedish study [7] and further modified by the study by Alqahtani RA et al. [3] to align with the cultural context of the Arabic-speaking region, was utilized in this study. The adaptation process involved linguistic and contextual modifications to ensure cultural relevance. The questionnaire was translated into Arabic by bilingual experts and subsequently back-translated to English to maintain accuracy. A panel of subject matter experts reviewed the instrument for content validity, ensuring that the items were appropriate and comprehensible for the target population. A pilot study was conducted with a sample of participants to assess clarity, cultural appropriateness, and feasibility. Based on participant feedback, minor refinements were made to enhance understanding and applicability.

The reliability and validity of the questionnaire were evaluated through a psychometric analysis. The internal consistency of the instrument demonstrated good reliability, with a Cronbach’s alpha exceeding 0.7 [3], indicating strong consistency among the items. Furthermore, construct validity was examined using exploratory factor analysis (EFA) to confirm the suitability of the questionnaire in the Arabic context.

The questionnaire consists of 31 items distributed across five sections:

Section I: Demographic characteristics of participants (4 items) - including age (in years), years of study, marital status, and nationality.

Section II: Perspectives on having children (4 items).

Section III: Factors affecting students’ decision to have children (7 items). Responses are based on a 5-point Likert scale (Strongly agree = 5, Agree = 4, Neutral = 3, Disagree = 2, Strongly disagree = 1), with a total score categorized as follows: Good: 35-23, Fair: 22-17 and Poor: 0-7.

Section IV: Students’ perspectives toward motherhood (9 items). Responses follow the same 5-point Likert scale, with the total score categorized as: Good: 45-31, Fair: 30-23, and Poor: 0-9

Section V: Students’ perspectives toward fertility issues (7 items). Each correct answer is scored as 1, and incorrect answers as 0. The total score classification is: Good: Maximum score of 7, Fair: 5-4, and Poor: 0-2.

The English version of the questionnaire was administered to non-Arabic-speaking participants in Turkey to ensure inclusivity in data collection.

Data collected from the study sample was revised, coded, and entered using a personal computer. Computerized data entry and statistical analysis were performed using SPSS version 22 (IBM Corp., Armonk, NY, US). Data were presented using descriptive statistics in the form of frequencies, percentages, and Mean ± SD.

Correlation coefficients were used to measure the strength of the relationship between two variables. Multiple linear regression (MLR), also known as multiple regression, is a statistical technique that uses several explanatory variables for predicting the outcome of a response variable. Chi-square test statistics were commonly used for testing relationships between categorical variables. Linear regression is a linear approach for modeling the relationship between a sculptor and one or more explanatory variables.

The significance of the results was determined as follows: highly significant at p value < 0.01, statistically significant at p value < 0.05, and nonsignificant at p value ≥ 0.05.

## Results

The study recruited 1,371 participants as the actual sample for this study. The profile distribution of students from the five colleges “Nursing, Medicine, Physiotherapy, and Medical Laboratory” were grouped by country. The study focused on the following parameters: socio-demographic characteristics, students' perspectives toward having children, factors affecting students’ decision to have children, students’ perspectives toward fertility concepts, students’ perspectives regarding oocyte cryopreservation, and the relationship between students’ characteristics and factors affecting students.

The following findings ratify the above parameters.

Table 1 lists the findings related to independent variables. More than half of the respondents (58.7%) were between 20 and 25 years old. Regarding nationality, more than one-quarter (28.4%) were from Iraq, 24.7% were from Egypt, 23% were from Turkey, and 2.7% were from Sudan. The majority (85.5%) were nursing students, while 3.7% were physiotherapy students. Most of the respondents (86.3%) were single. Almost half (47%) had previous information regarding fertility in general, and about one-third (38%) obtained this information from friends.

**Table 1:** Socio-demographic characteristics of the respondents (N = 1,371) Chi-square test (χ² test) # Not mutually exclusive

Items		n	%
Age:	< 20 years	464	33.9
	20–25 years.	805	58.7
	26–30 years	76	5.5
	>30 years	26	1.9
	Mean + SD	21.46 + 3.57	
Nationality:	Iraq	390	28.4
	Egypt	338	24.7
	Turkey	316	23
	Jordan	168	12.3
	Saudi	122	8.9
	Sudan	37	2.7
Specialty:	Nursing	1,171	85.5
	Medical science	73	5.3
	Medicine	52	3.9
	Physiotherapy	50	3.7
	Medical laboratory	21	1.6
Marital status:	Single	1,183	86.3
	Married	172	12.6
	Divorced	14	1
	Widowed	2	0.1
Previous information regarding fertility:	Yes	645	47
	Somewhat	538	39.4
	No	188	13.6
Source of your information = 1,183 #	Reading resources (books, study, scientific website)	390	33
	Friends	450	38.0
	Family	310	26.2
	Social media	200	16.9

Table 2 presents the perspectives of the studied students (dependent variables) on having children. The majority (83.7%) expressed a desire to have children in the future, while 3.9% did not wish to have children. Regarding the desired number of children, 82.4% of respondents reported wanting 1-3 children, whereas 1.5% reported wanting more than 6 children. Concerning the preferred age for having the first child, the majority (91.3%) indicated an age range of 20-30 years old.

**Table 2:** Perspectives of students from multi-country backgrounds toward having children (N = 1,371)

Items	n	%
Have children in the future		
Yes	1,147	83.7
No	53	3.9
Not sure	171	12.4
Frequency of children you desire		
1-3 children	1,130	82.4
4-6 children	221	16.1
More than six children	20	1.5
Desired age at first child		
<20 years	25	1.8
20–30 years	1,252	91.3
>30 years	94	6.9

Table 3 lists the factors affecting students’ decisions to have children. More than one-third (39.6%) strongly agreed with having children after completing their education, while 2.7% strongly disagreed. Two-fifths (40%) agreed with the statement “if they could work and have children at the same time.” Almost half (48.4%) agreed with having children if they had access to childcare, while 3.5% strongly disagreed. More than one-third (40.8%) agreed with having children after securing a permanent position. About 45.4% agreed with having children before becoming” too old.” Almost half (48%) of respondents strongly agreed with having children after making progress in their careers.

**Table 3:** Factors affecting the decisions of students from multi-country backgrounds regarding having children (N = 1,371)

Condition	Strongly agree		Agree		Neutral		Disagree		Strongly disagree	
	n	%	n	%	n	%	N	%	n	%
Have children after completing my studies	543	39.6	535	39	195	14.3	61	4.4	37	2.7
Work and have children simultaneously	98	7	577	42	351	25.5	223	16.6	122	8.9
Have children if I can access childcare	188	13.7	664	48.4	322	23.5	149	10.9	48	3.5
Have children after getting a permanent position	242	17.7	559	40.8	343	25	234	17	48	3.5
Have children before becoming “too old”	516	37.7	622	45.4	128	9.3	69	5	36	2.6
Have children after I have had time to travel and do other things difficult to do with children	336	24.5	416	30.3	301	22	252	18.4	66	4.8
Have children after I advance in the profession	659	48	543	39.5	100	7.3	23	1.7	20	1.5

**Figure 1:**
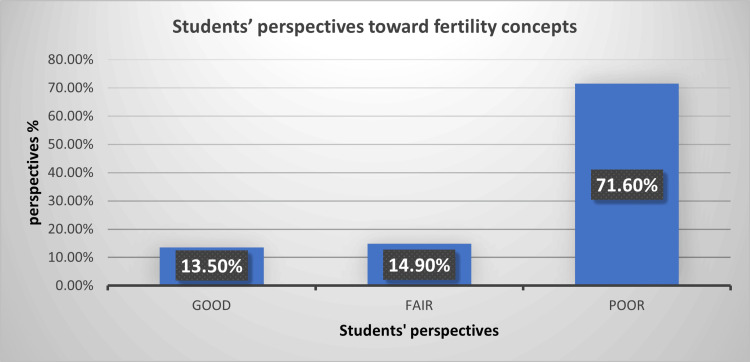
Perspectives of students from multi-country backgrounds toward fertility concepts (n = 1,371)

Figure 1 shows the overall perspectives of the studied students toward fertility concepts. Nearly three-quarters (71.6%) had poor perspectives, 14.9% had fair perspectives, and 13.5% had good perspectives. The categories of knowledge were based on students’ answers, with knowledge levels evaluated as follows: 0-2 correct answers indicate severe gaps in knowledge about fertility, 3-4 correct answers indicate solid basic knowledge, and 5-7 correct answers indicate good knowledge about fertility.

**Figure 2:**
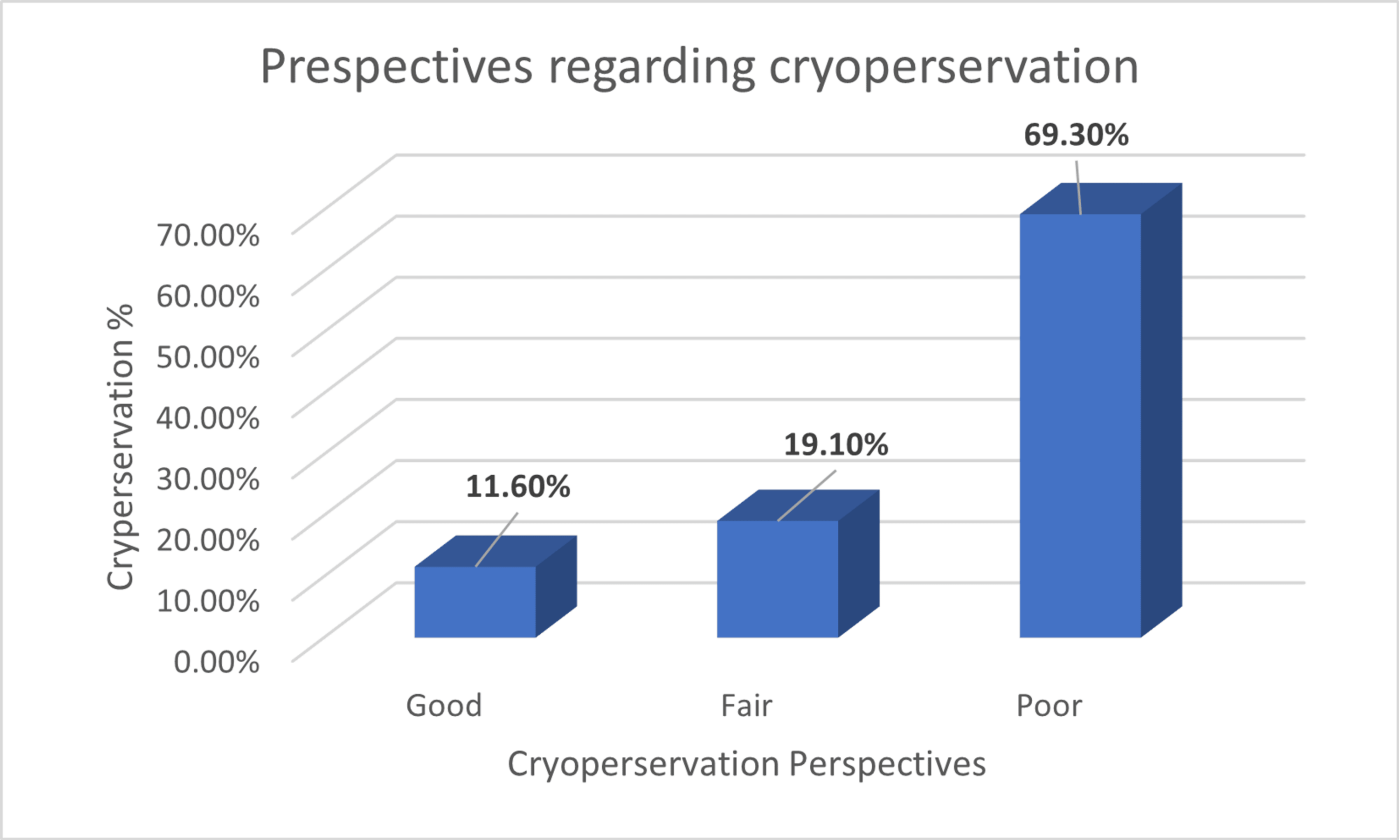
Perspectives of students from multi-country backgrounds toward oocyte cryopreservation (N = 1,371)

Figure 2 shows that more than two-thirds of students (69.3%) had poor perspectives toward oocyte cryopreservation due to limited knowledge. Additionally, 19.1% had fair perspectives, while 11.6% had good perspectives. The knowledge categories based on students’ answers were as follows: 0-2 correct answers indicate severe gaps in knowledge about fertility, 3-4 correct answers indicate solid basic knowledge, and 5-7 correct answers indicate good knowledge about fertility.

Table 4 shows the chi-square test (χ² test) with highly statistically significant relationships between students’ perspectives (dependent variable) regarding oocyte cryopreservation and independent variables, including age, specialty, marriage, and previous knowledge about fertility, indicated by chi-square tests of 4.998 (P < 0.05*), 6.002 (P < 0.05*), and 14.073 (P < 0.01*), respectively. However, no statistically significant relationships were found between perspectives and nationality (χ²= 1.075, P > 0.05) or marital status (χ²= 1.042, P > 0.05).

**Table 4:** Relationship between students’ characteristics and their perspectives toward oocyte cryopreservation (N = 1,371) Chi-square test (χ² test) *Slight significant <0.05*; **high significant if p value <0.01**

Items		Perspectives regarding oocyte cryopreservation						Chi-square	P value
		Good (159)		Fair (262)		Poor (950)			
	N	n	%	n	%	n	%		
Age:									
< 20 years	464	30	6.5	90	19.4	344	74.1	4.998	<0.05*
20–25 years	805	64	7.9	146	18.1	595	73.9		
26–30 years	76	45	59.2	21	27.6	10	13.2		
>30 years	26	20	76.9	5	19.3	1	3.8		
Nationality:									
Iraq	390	45	11.6	70	17.9	275	70.5	1.075	>0.05
Egypt	338	42	12.4	76	22.5	220	65.1		
Jordan	168	17	10.1	20	11.9	131	78		
Saudi	122	15	12.3	16	13.1	91	74.6		
Sudan	37	6	16.2	4	10.8	27	73		
Turkey	316	34	10.7	76	24.1	206	65.2		
Specialty:									
Medicine	52	50	96.2	2	3.8	0	0		
Nursing	1,171	33	2.8	235	20.1	907	77.1	6.002	<0.05*
Physiotherapy	50	7	14	6	12	37	74		
Medical laboratory	21	16	76.2	4	19	1	4.8		
Medical science	73	53	72.6	15	20.5	5	6.9		
Marital status:									
Married	172	40	23.3	82	47.7	50	29	1.042	>0.05
Single	1,183	114	9.6	174	14.7	895	75.7		
Divorced	14	4	28.6	5	35.7	5	35.7		
Widowed	2	1	50	1	50	0	0		
Previous information on fertility:								14.073	<0.01**
Yes	645	125	19.4	220	34.1	300	46.5		
Somewhat	538	25	4.6	30	5.6	483	89.8		
No	188	9	4.8	12	6.4	167	88.8		

In Table 5, the chi-square test (χ² test) shows significant relationships between students’ characteristics (independent variables) and their perspectives (dependent variable) toward motherhood. Particularly, there are statistically significant relationships between respondents’ age and their previous information about fertility, indicated by χ² values of 13.404 (P < 0.01**) and 9.886 (P < 0.01**), respectively. However, no statistically significant relationships were found between respondents’ nationality, specialty, or marital status and their perspectives toward motherhood, as evidenced by χ² values of 1.098 (P > 0.05), 2.009 (P > 0.05), and 6.005 (P > 0.05), respectively.

**Table 5:** Correlations between students’ decisions to have children and their perspectives toward motherhood Chi-square test (χ² test) *Slight significant <0.05*; **high significant if p value <0.01**

Items		Perspective toward motherhood						Chi-square	P value
		Good (650)		Fair (502)		Poor (219)			
	N	n	%	n	%	n	%		
Age:									
< 20 years	464	69	14.9	270	58.2	125	27	13.404	0.01**
20–25 years	805	499	62	216	26.8	89	11		
26–30 years	76	60	79	12	15.8	5	6.6		
>30 years	26	22	84.6	4	15.4	0	0		
Nationality:									
Iraq	390	172	44.1	127	32.6	91	23.3	1.098	0.05
Egypt	338	150	44.8	135	40	53	15.7		
Jordan	168	87	51.7	61	36.3	20	12		
Saudi	122	60	49.2	62	50.8	0	0		
Sudan	37	15	40.5	7	19	15	40.5		
Turkey	316	166	52.5	110	34.8	40	12.7		
Specialty:									
Medicine	52	26	50	19	36.5	7	13.5	2.009	0.05
Nursing	1,171	575	49.1	447	38.2	149	12.7		
Physiotherapy	50	23	46	10	20	17	34		
Laboratory	21	11	52.4	4	19	6	28.6		
Medical science	73	24	32.9	9	12.3	40	54.8		
Marital status:									
Married	172	160	93	12	7	0	0	6.005	0.05
Single	1,183	488	41.3	487	41.1	208	17.6		
Divorced	14	2	14.3	2	14.3	10	71.4		
Widowed	2	0	0	1	50	1	50		
Previous information on fertility:									
Yes	645	510	79	116	18	19	3	9.886	0.01**
Somewhat	538	120	22.3	368	68.4	50	9.3		
No	188	20	10.6	18	9.6	150	79.8		

In Table 6, Pearson's correlation coefficient (r) provides a strong positive correlation between the independent variables “students’ decision to have children” and the dependent variables “students’ perspectives” regarding cryopreservation and motherhood, as indicated by r = 0.527 (P < 0.01**), r = 0.533 (P < 0.01**), and r = 0.406 (P < 0.01**), respectively.

**Table 6:** Correlations between studied students’ decision to have children’s factors, cryopreservation. Pearson's correlation coefficient (r) *Slight significant <0.05* **high significant if p value <0.01**

		1	2	3	4
1-Factors affecting students’ decision to have children	r.		0.527	0.580	0.499
	p.		<0.01**	<0.01**	<0.01**
2-Perspectives toward cryopreservation	r.			0.533	0.470
	p			<0.01**	<0.01**
3-Prespective toward motherhood	r.				0.406
	p.				<0.01**

## Discussion

The findings of this study determined students’ perspectives toward fertility issues in six different countries. Based on the analysis of the findings, most student respondents were 20-25 years old. Almost half of them have previous information regarding fertility, and about one-third of them get information from friends.

In the current study, most participants desired to have children in the future, while only 3.9% did not. Similarly, previous studies conducted in Arab countries [2,3] about fertility awareness among health professional students showed that most healthcare profession students have a clear desire to have children, as most in different European countries [1,18,20,26]. Based on these findings, there is no difference between European and Arab cultures in the idea of desiring to have children, which may be related to having no restrictions in these countries to have children. However, these findings are dissimilar to a study conducted among Chinese students who expressed an abundant decline in the desire to have children, which is related to the characteristics of urban culture that prefer to secure better education and work first [27].

Regarding the number of children they desire, the majority of participants reported 1-3 children, while the least reported more than six children. As regards the desired age at first child, most of them mentioned 20-30 years old. These findings match those of previous studies in Iraq, Saudi Arabia, and Egypt [1,2,13], reflecting that the ideal number for young females was almost three children and a smaller percentage intended to have more than three children, which is also in line with the average number of two children reported by previous European studies from Turkey, Denmark, and Ukraine [1,18,26]. The similarity between Arab and European countries is due to the similarity in the participants’ backgrounds and age groups.

The factors affecting students’ decision to have children based on the findings of the current study revealed that most of the participants agreed to have children after completing their studies. One-fourth disagreed with working and having children simultaneously. Almost two-thirds of them agreed with having children if they had access to childcare as well as having children after securing a permanent position, and almost half of them agreed with having children before they were “too old” and after achieving a promotion in their profession. Similarly, the previous studies expected that their motherhood would affect their studies and careers; therefore, the Saudi study confirmed that the participants preferred to have children after having secured an advanced career position [3]. Along the same lines, Turkish students are more concerned about their career and work life being secured first [18]. Likewise, participants in the Danish study preferred to complete their education, start a career, and be in a permanent job before having children [1]. The similarities between the findings from different cultures reflected the importance of women’s role in society, in addition to the main responsibility of childcare, which was conducted mostly by females.

There is a significant relationship between students’ characteristics and their perspectives toward motherhood based on their age and previous information about fertility, while there is no statistically significant relation found based on nationality and marital status, reflecting that the main variable of variation between the participants is based on age and only previous information. However, the variation of the nationality from six countries has no effect in the current study, and this is in line with previous studies, which confirmed that age was always an important factor related to motherhood [1,18,27].

The current study asked the participating students about their perspectives toward oocyte cryopreservation as one of the fertility preservation strategies; the findings reflected that most of them have poor awareness, while only 10.70% of them had good awareness. Also, more than three-quarters (77%) had a negative attitude. The participants’ age, previous information about fertility, and their specialty reflected a significant relation (p = <0.01**). The negative attitude in the current study toward cryopreservation is congruent with the reported findings from previous studies, reflecting that most undergraduate medical students would not consider elective oocyte preservation at a young age [28-30].

The conclusion of all studied variables reflected a positive correlation between participants’ decision to have children and their perspectives regarding cryopreservation and motherhood with a high-frequency positive effect on students’ perspectives of fertility issues.

Limitations

The study includes a diverse range of cultures; however, the demographic data focused solely on the relationship between students' age and their perspectives, without considering socioeconomic, religious, or cultural influences on adolescent viewpoints. Additionally, the reliance on convenience sampling limits the generalizability of the findings. Furthermore, the results presented the total number of participants for each variable without specifying the findings for each country.

## Conclusions

This study presents the different demographic characteristics of the participants, showing that these factors did not affect the findings. It also reflects that almost half of the participants from each country have a positive perspective on motherhood and a desire to have children in the future, typically between the ages of 20 and 30. However, there are poor perspectives on fertility concepts and oocyte cryopreservation across different countries. Therefore, the study highlights the need to increase students’ knowledge and awareness of fertility and related issues during this stage of life. The study recommends that nurses provide the necessary informed guidance and support to young females and males who are making fertility-related decisions. Additionally, emphasizing the potential impact of motherhood on students’ perspectives regarding fertility issues can enhance their preparedness to address these concerns in their future practice.
